# Down-regulation of the caffeic acid *O*-methyltransferase gene in switchgrass reveals a novel monolignol analog

**DOI:** 10.1186/1754-6834-5-71

**Published:** 2012-09-21

**Authors:** Timothy J Tschaplinski, Robert F Standaert, Nancy L Engle, Madhavi Z Martin, Amandeep K Sangha, Jerry M Parks, Jeremy C Smith, Reichel Samuel, Nan Jiang, Yunqiao Pu, Arthur J Ragauskas, Choo Y Hamilton, Chunxiang Fu, Zeng-Yu Wang, Brian H Davison, Richard A Dixon, Jonathan R Mielenz

**Affiliations:** 1 Biosciences Division, Oak Ridge National Laboratory, Oak Ridge, TN, 37831-6341, USA; 2 School of Chemistry and Biochemistry, Georgia Institute of Technology, Atlanta, GA, 30332, USA; 3 Forage Improvement Division, The Samuel Roberts Noble Foundation, 2510 Sam Noble Parkway, Ardmore, OK, 73401, USA; 4 Plant Biology Division, The Samuel Roberts Noble Foundation, 2510 Sam Noble Parkway, Ardmore, OK, 73401, USA; 5 BioEnergy Science Center, Oak Ridge, TN 38731, USA; 6 Department of Biochemistry and Molecular & Cellular Biology, University of Tennessee, Knoxville, TN, 37996, USA

**Keywords:** *trans*-3, 4-Dimethoxy-5-hydroxycinnamyl alcohol, *iso*-Sinapyl alcohol, Monolignol, Switchgrass, Bioenergy, Recalcitrance, Caffeic acid *O*-methyltransferase, Transgenic

## Abstract

**Background:**

Down-regulation of the caffeic acid 3-*O*-methyltransferase EC 2.1.1.68 (COMT) gene in the lignin biosynthetic pathway of switchgrass (*Panicum virgatum*) resulted in cell walls of transgenic plants releasing more constituent sugars after pretreatment by dilute acid and treatment with glycosyl hydrolases from an added enzyme preparation and from *Clostridium thermocellum*. Fermentation of both wild-type and transgenic switchgrass after milder hot water pretreatment with no water washing showed that only the transgenic switchgrass inhibited *C. thermocellum*. Gas chromatography–mass spectrometry (GCMS)-based metabolomics were undertaken on cell wall aqueous extracts to determine the nature of the microbial inhibitors.

**Results:**

GCMS confirmed the increased concentration of a number of phenolic acids and aldehydes that are known inhibitors of microbial fermentation. Metabolomic analyses of the transgenic biomass additionally revealed the presence of a novel monolignol-like metabolite, identified as *trans*-3, 4-dimethoxy-5-hydroxycinnamyl alcohol (*iso*-sinapyl alcohol) in both non-pretreated, as well as hot water pretreated samples. *iso*-Sinapyl alcohol and its glucoside were subsequently generated by organic synthesis and the identity of natural and synthetic materials were confirmed by mass spectrometric and NMR analyses. The additional novel presence of *iso*-sinapic acid, *iso*-sinapyl aldehyde, and *iso*-syringin suggest the increased activity of a *para*-methyltransferase, concomitant with the reduced COMT activity, a strict *meta*-methyltransferase. Quantum chemical calculations were used to predict the most likely homodimeric lignans generated from dehydration reactions, but these products were not evident in plant samples.

**Conclusions:**

Down-regulation of COMT activity in switchgrass resulted in the accumulation of previously undetected metabolites resembling sinapyl alcohol and its related metabolites, but that are derived from *para*-methylation of 5-hydroxyconiferyl alcohol, and related precursors and products; the accumulation of which suggests altered metabolism of 5-hydroxyconiferyl alcohol in switchgrass. Given that there was no indication that *iso*-sinapyl alcohol was integrated in cell walls, it is considered a monolignol analog. Diversion of substrates from sinapyl alcohol to free *iso*-sinapyl alcohol, its glucoside, and associated upstream lignin pathway changes, including increased phenolic aldehydes and acids, are together associated with more facile cell wall deconstruction, and to the observed inhibitory effect on microbial growth. However, *iso*-sinapyl alcohol and *iso*-sinapic acid, added separately to media, were not inhibitory to *C. thermocellum* cultures.

## Background

There are three well known monolignol precursors that polymerize to form the lignin that binds plant cell walls together: *p*-coumaryl alcohol, coniferyl alcohol, and sinapyl alcohol. These result, respectively, in the hydroxyphenyl (H), guaiacyl (G), and syringyl (S) monomer units of the lignin polymer. The relative proportion of monolignols can determine the ease of cell wall deconstruction by enzymatic or biocatalyst-mediated mechanisms [1]. For example, the ratio of S to G residues, the most abundant monolignols in angiosperms, can impact the degree of cross-linking of the lignin, the degree of condensation, and hence, the spatial arrangement and accessibility of the lignin to deconstruction [2-4]. The polymerization of these monolignols and their cross-linking with phenolic acids to hemicellulosic sugars are keys to the recalcitrance of cell walls to enzymatic hydrolysis that is required to release sugars for biofuel production. High S/G ratios are considered favorable for deconstruction in angiosperms [5], but the reverse is true for alfalfa, tall fescue and switchgrass [1,6,7]. In some cases, lignin content appears to be more predictive of recalcitrance than does lignin composition [1]. The contents of lignin and ether-bound phenolics in the cell wall were the major determinants of the biomass degradation caused by enzymatic hydrolysis in *Miscanthus* genotypes [8]. Other studies also suggest that either lignin content or composition can play a role in sugar release from cell walls of grasses and trees, including *Miscanthus*[9] and *Populus*[10].

Down-regulation of the caffeic acid 3-*O*-methyltransferase EC 2.1.1.68 (COMT) gene in the lignin biosynthetic pathway of switchgrass (*Panicum virgatum*) produced transgenic plants with a normal growth phenotype, but with reduced lignin content, altered lignin composition, improved forage quality, increased saccharification efficiency, and increased ethanol production yield from the modified substrate compared with the controls [7]. Two of the COMT-deficient lines from this study had greatly reduced COMT expression levels versus the wild-type genetic background. Interestingly, there was a decline in the S/G ratio of cell walls of stems from 0.90 to 0.57, with S-lignin specifically reduced by up to 53%, and an overall decline in acetyl bromide lignin content of 12-14%, depending on the transgenic line. These responses imply a reduction of *trans*-sinapyl alcohol in these transgenic lines. Whereas the down-regulation of COMT may or may not result in a reduction in lignin content, it generally results in a reduction of S units in a variety of plant species lignin, including hybrid poplar (*Populus tremula x alba*) [11], alfalfa (*Medicago sativa*) [12], maize (*Zea mays*) [13], *Arabidopsis thaliana*[14], and tall fescue (*Festuca arundinacea*) [15]. Whereas S-units are typically reduced, G units may also be reduced, but to a lesser extent, thereby still resulting in the often reported increase in the S/G ratio of the lignin. Such a coupled reduction in both S and G units in response to down-regulation of COMT was observed in alfalfa [12] and in perennial ryegrass (*Lolium perenne*) [16]. Also frequently observed is a concomitant increase in the precursor 5-hydroxyguaiacyl units that are derived from the incorporation of 5-hydroxyconiferyl alcohol into transgenic lignin, as reported for the brown-rib mutant (*bmr3*) in maize with reduced COMT activity [17], in COMT-deficient hybrid poplar [18], and in the *Arabidopsis Atomt1* mutant [14,19]. Given such responses, broad effects on the metabolic network beyond the targeted transgenic manipulation should be expected.

Curiously, the COMT-deficient switchgrass residues that remain after mild pretreatment inhibit fermentation by the bacterium *Clostridium thermocellum*, compared to the wild-type switchgrass plants. Given that the COMT-deficient lines contain a genetic block in the lignin pathway [7], it was hypothesized that these plants have a reduced concentration of sinapyl alcohol and contain increased concentrations of phenolic aldehydes and acids related to the lignin biosynthetic pathway that are inhibitory molecules for biological processes [20]. We used gas chromatography–mass spectrometry (GCMS)-based metabolomic profiling of pretreated (hot water) biomass of down-regulated COMT switchgrass (*Panicum virgatum*) lines to reveal the greater presence of such inhibitory phenolic metabolites, and, in particular, a novel monolignol-like metabolite identified as *trans*-3, 4-dimethoxy-5-hydroxycinnamyl alcohol (*iso*-sinapyl alcohol) and related metabolites that accumulate in transgenic COMT-deficient switchgrass lines. The general consequences of the present findings for consolidated bioprocessing and switchgrass engineering for biofuel production are discussed.

## Results

### Bacterial fermentation of transgenic COMT-deficient versus wild-type switchgrass

In contrast to the previously mentioned published research with acid-soaked, pretreated switchgrass, which requires washing to remove acid, the unwashed water-pretreated transgenic switchgrass solids failed to ferment fully, compared to the wild-type switchgrass. These results come from experiments comparing the impact of milder pretreatment conditions on the transgenic COMT down-regulated and wild-type switchgrass variety ‘Alamo’, used previously with more severe pretreatment [7], in conjunction with fermentation by *C. thermocellum*. Pretreatment was conducted on water-soaked switchgrass at 180°C for 25 minutes. Specifically, the wild-type yielded total fermentation products (lactic acid, acetic acid, ethanol) at 208.1 ± 2.8 mg total products/g cellulose, while the transgenic COMT-deficient switchgrass, which was the same line used [7], had essentially the same yield on substrate of 196.8 ± 20.5 mg total products/g cellulose. These results were unexpected as the same samples had yielded up to 38% more ethanol per gram cellulose for the COMT transgenic switchgrass vs. the wild-type biomass, using a yeast-based simultaneous saccharification and fermentation process with the washed, acid pretreated samples. The reduced yield by the transgenic switchgrass suggested an inhibition of fermentation not seen with previous samples of the free liquid available after hot water pretreatment.

### Metabolomic profiles of hydrolysates of COMT down-regulated versus wild-type switchgrass

There were unexpected responses in metabolomic profiles resulting from GCMS-based analyses of aqueous extracts of the mild water pretreated biomass of transgenic COMT down-regulated and wild-type switchgrass variety ‘Alamo’. The key changes in metabolomic profiles resulting from the down-regulation of COMT are shown in Table 1. Responses that are unrelated to the lignin biosynthetic pathway, but were nonetheless outstanding in COMT down-regulated plants, included the accumulation of purine bases and their corresponding nucleosides. For example, a number of purines and pyrimidines, including adenine, guanine, uracil, hypoxanthine and xanthine, were increased 1.4- to 2.7-fold. Associated nucleosides, including uridine and guanosine, were also elevated 1.6 to 2.4-fold, respectively, but adenosine was unchanged. Several organic acids, including maleic, citraconic, and succinic acids, were similarly increased 1.6-2.6-fold. Such unexpected responses in pathways remote from the targeted pathway can be challenging to explain.

**Table 1:** Metabolite concentrations [mean (sem)] and fold change of down-regulated COMT versus wild-type (WT) switchgrass

**Metabolite**	**COMT concentration (μg/ml)**	**WT concentration (μg/ml)**	**COMT/WT fold change**	**P-value**
*iso*-sinapyl alcohol	0.83 (0.06)	nd	∞	0.000
*iso*-sinapic acid	0.11 (0.01)	nd	∞	0.000
*15.09 354 239 620 lignan*^a^	0.34 (0.02)	nd	∞	0.000
*15.18 354 219 M + 530 (iso-sinapyl-phenolic)*	0.11 (0.01)	nd	∞	0.000
5-hydroxyconiferyl alcohol-4-*O*-glucoside	0.08 (0.01)	0.00 (0.00)	76.05	0.000
5-hydroxyconiferyl alcohol-5-*O*-glucoside	0.17 (0.01)	0.00 (0.00)	59.83	0.000
*iso*-syringin	2.60 (0.32)	0.06 (0.01)	42.79	0.000
*12.37 428 327 209 413*	0.39 (0.02)	0.03 (0.01)	14.09	0.000
3,4-dihydroxybenzoic acid	7.52 (3.72)	1.19 (0.34)	6.34	0.096
xanthine	1.14 (0.14)	0.43 (0.18)	2.68	0.019
hypoxanthine	2.85 (0.96)	1.07 (0.17)	2.65	0.080
succinic acid	14.98 (3.97)	5.78 (1.04)	2.59	0.041
guanosine	5.18 (0.76)	2.17 (0.62)	2.39	0.018
uracil	2.02 (0.29)	0.98 (0.22)	2.07	0.023
citraconic acid	2.25 (0.18)	1.23 (0.31)	1.82	0.033
guanine	7.24 (0.28)	4.10 (0.85)	1.77	0.016
5-hydroxyferulic acid	0.46 (0.01)	0.27 (0.02)	1.69	0.000
uridine	11.86 (0.49)	7.27 (1.39)	1.63	0.026
maleic acid	65.92 (1.63)	41.74 (7.23)	1.58	0.023
vanillin	26.20 (1.63)	16.81 (3.48)	1.56	0.060
secoisolariciresinol	2.27 (0.13)	1.47 (0.38)	1.54	ns
5-*oxo*-proline	116.72 (6.82)	77.40 (7.46)	1.51	0.007
adenine	10.19 (0.72)	7.25 (0.91)	1.41	0.045
1-*O-trans-*feruloylglycerol	1.05 (0.05)	0.77 (0.11)	1.37	0.065
ferulic acid	7.06 (0.17)	5.50 (0.14)	1.28	0.000
*5-hydroxyconiferaldehyde (13.08 M + 338 323 )*	0.26 (0.02)	0.20 (0.01)	1.28	0.029
adenosine	9.62 (0.66)	7.58 (0.86)	1.27	ns
*p*-coumaric acid	21.80 (0.21)	19.31 (0.57)	1.13	0.08
caffeic acid	0.58 (0.04)	0.53 (0.04)	1.11	ns
*p*-hydroxybenzaldehyde	4.63 (0.33)	4.24 (0.80)	1.09	ns
coniferyl alcohol	3.34 (0.10)	3.65 (0.22)	0.92	ns
5-hydroxyconiferyl alcohol	1.22 (0.06)	1.36 (0.08)	0.90	ns
coniferyl aldehyde	0.35 (0.01)	0.45 (0.07)	0.78	ns
guaiacylglycerol	5.25 (0.23)	6.79 (0.53)	0.77	0.047
sinapyl aldehyde	0.17 (0.02)	0.22 (0.03)	0.77	ns
syringin	0.23 (0.01)	0.30 (0.03)	0.77	0.093
sinapyl alcohol	2.51 (0.06)	3.48 (0.20)	0.72	0.004
syringylglycerol	2.99 (0.09)	4.48 (0.25)	0.67	0.001
*p*-hydroxyphenylpyruvic acid	0.63 (0.05)	0.96 (0.09)	0.65	0.021
*guaiacylglycerol glycoside (14.51 297 608 593)*	3.53 (0.17)	5.57 (0.69)	0.63	0.038
syringaresinol	0.07 (0.00)	0.14 (0.01)	0.52	0.001
*guaiacylglycerol glycoside (14.57 297 608 593)*	3.59 (0.19)	5.98 (0.88)	0.60	0.050
*syringylglycerol glycoside (16.12 327 361 239)*	3.91 (0.22)	7.88 (1.34)	0.50	0.035
pinoresinol	0.22 (0.03)	0.45 (0.07)	0.49	0.031
*guaiacylglycerol glycoside (15.87 297 361 323)*	7.42 (0.30)	17.96 (4.41)	0.41	0.073
hydroxymethylfurfural	0.72 (0.12)	3.40 (1.41)	0.21	ns

^a^ Metabolites in italics are tentative identifications.

The fundamental target of reducing the production of *trans*-sinapyl alcohol was achieved as expected by down regulation of the caffeic acid 3-*O*-methyltransferase, with the concentration in the transgenic lines reduced to 72% of that of the wild-type control. This was confirmed by similar reductions in related metabolites, including the conjugation product syringin (sinapyl alcohol glucoside; 77%), syringaresinol (52%), a lignan, and syringylglycerol (67%), a related wall degradation catabolite, and its glycoside (50%). Another abundant wall degradation catabolite, guaiacylglycerol, and glycosidic conjugates were similarly reduced in COMT down-regulated plants. These latter metabolites are, however, related to coniferyl alcohol, the other major monolignol precursor. Pinoresinol, a lignan of coniferyl alcohol, was also reduced to 49% that observed in the wild-type lines. These responses contrast with increases in the major phenolic aldehyde related to coniferyl alcohol, vanillin that increased 1.56-fold. Similarly, phenolic acids related to coniferyl alcohol, including ferulic acid and 5-hydroxyferulic acid, were also increased 1.3- and 1.7-fold, respectively, and 1-*O-trans*-feruloylglycerol was also elevated 1.4-fold. An unknown compound eluting at 15.18 min (338 354 mass-to-charge ratio; m/z) that shares m/z with ferulic acid, and hence may be a conjugate, was evident only in transgenic plants. Another unidentified lignan (RT 15.09 min, 239 354 620 m/z) was also only evident in COMT down-regulated plants. Many of these phenolic aldehydes, acids, and lignans are major microbial growth and fermentation inhibitors. Despite the increases in coniferyl alcohol-related phenolic aldehydes and acids, and declines in the aforementioned related wall metabolites, the monolignols, coniferyl alcohol and 5-hydroxyconiferyl alcohol, and upstream phenolic acid precursors, including *p*-coumaric acid and caffeic acid were unchanged (which contrasts with the decline in sinapyl alcohol). Although 5-hydroxyconiferyl alcohol was unchanged, its precursor, 5-hydroxyconiferaldehyde was increased 1.28-fold, and its 4-*O*- and 5-*O*-glucosides were 76-fold and 60-fold higher, respectively, in the COMT down-regulated lines, with concentrations albeit low for even these plants.

### Identification of iso-sinapyl alcohol and its glucoside

**Figure 1:**
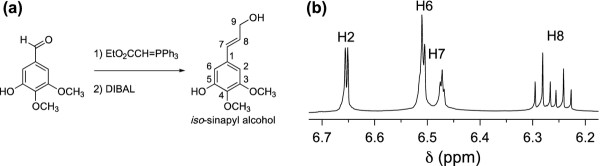
**(a) Synthesis of *trans*-3,4-dimethoxy-5-hydroxycinnamyl alcohol (*iso*-sinapyl alcohol).** (**b**) ^1^ H NMR spectrum of synthetic *iso*-sinapyl alcohol (aromatic and double-bond region).

**Figure 2:**
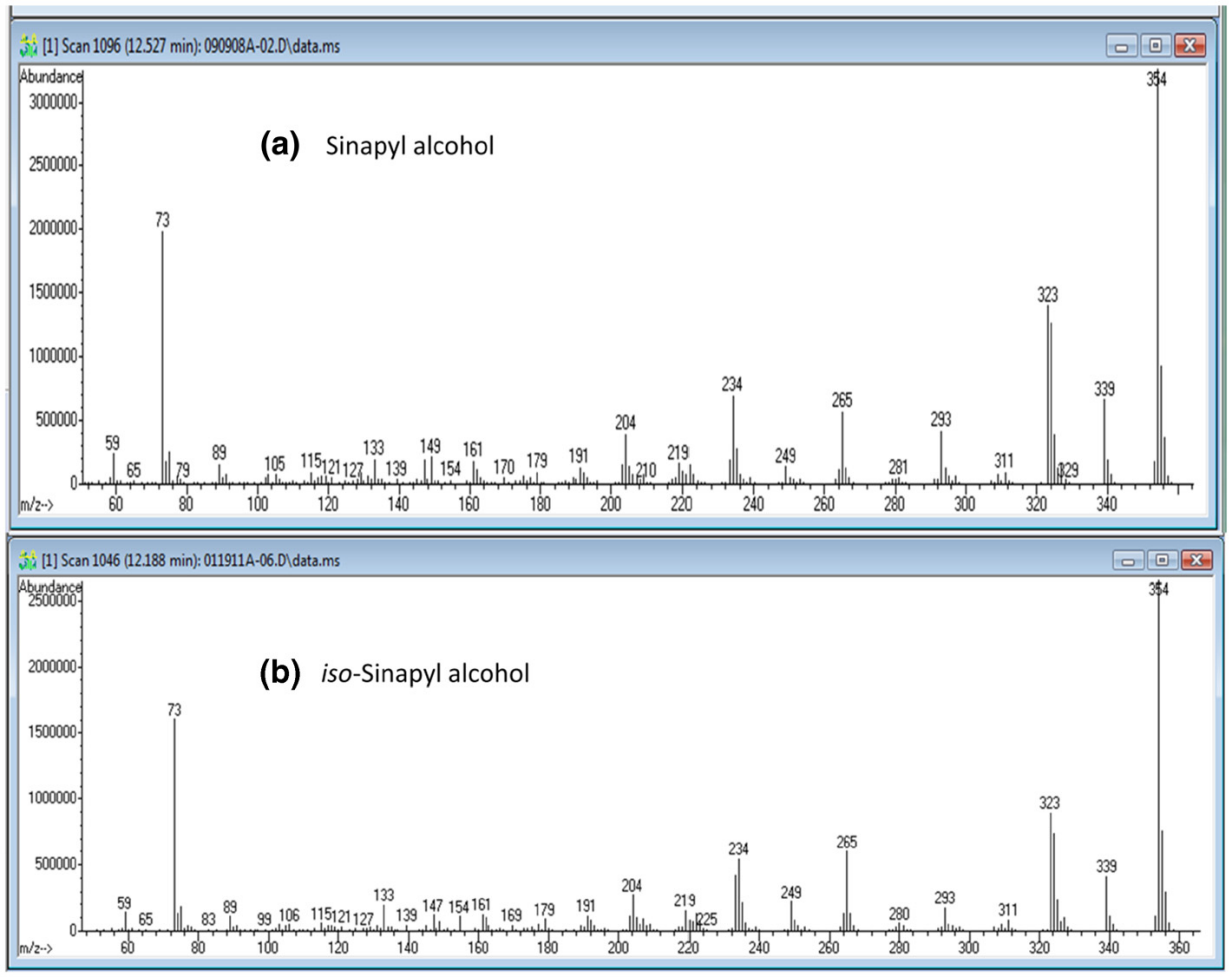
**GCMS EI fragmentation pattern of trimethylsilyl derivatized a) sinapyl alcohol and synthetic b) *iso*-sinapyl alcohol.**

**Figure 3:**
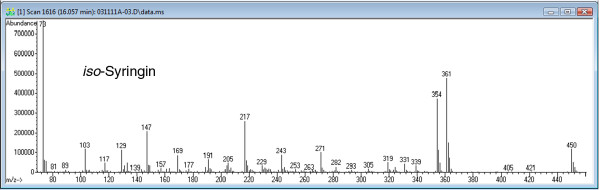
**GCMS electron ionization (70 eV) fragmentation pattern of trimethylsilyl derivatized synthetic3, 4-dimethoxy-5-hydroxycinnamyl alcohol-5**-***O*-glucoside (*iso*-syringin).**

**Figure 4:**
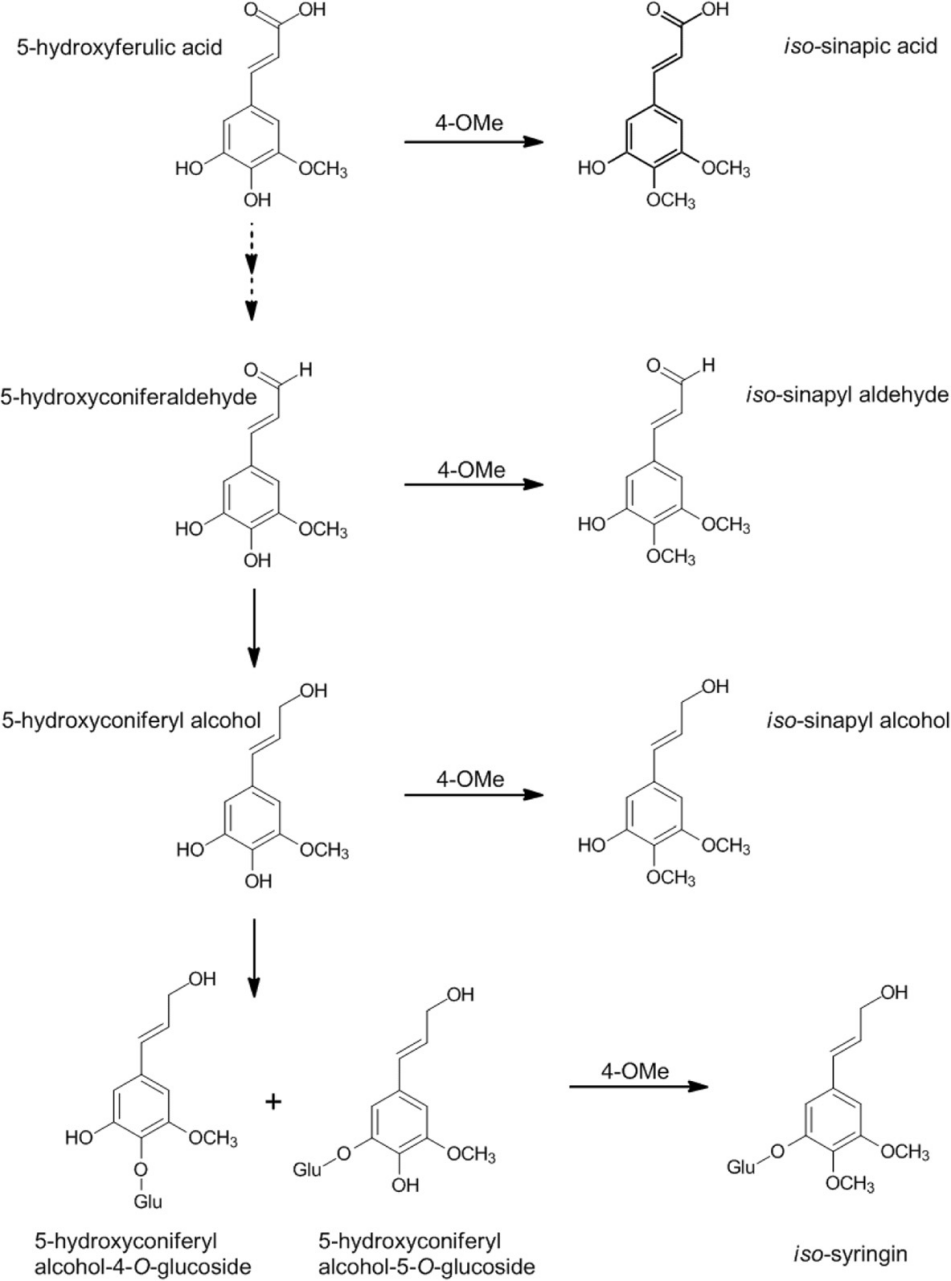
**The structures of the observed *iso*-sinapyl alcohol related metabolites and potential synthesis routes.**

The transgenic COMT down-regulated samples additionally accumulated two novel peaks that resembled sinapyl alcohol and its 4-*O*-glucoside, syringin, but both peaks eluted earlier than expected. Analysis with a Waters GCT Premier accurate mass gas chromatograph-time-of-flight-mass spectrometer indicated that the molecular formula of the unknown monomer was identical to that of sinapyl alcohol. Generation of the *cis*-isomer following a 24 h exposure of a *trans*-sinapyl alcohol commercial standard to UV-light (254 nm) confirmed that the sinapyl alcohol-like peak was not simply the *cis*-isomer of the normally observed *trans*-metabolite, with the *cis*-isomer eluting earlier than the unidentified peak. Given the relative retention time (RT) of the unknown peak, it was hypothesized that the monomer was methylated at the O-4 position on the phenyl ring, instead of the O-5 position, as in sinapyl alcohol. To test this hypothesis, the isomeric monolignol was synthesized. The two-step synthesis (Figure 1a) involved a Wittig reaction between 3, 4-dimethoxy-5-hydroxybenzaldehyde and carbethoxymethylene triphenylphosphorane, followed by reduction of the resulting cinnamate ester with diisobutylaluminum hydride (DIBAL), as described by [21]. The product is named 3, 4-dimethoxy-5-hydroxycinnamyl alcohol (or simply *iso*-sinapyl alcohol). NMR (Figure 2b) confirmed the structure of the synthetic material, and its GCMS fragmentation pattern and RT (Figure 2) both confirmed the identity of the unknown monomer and the synthetic product. Additionally, we synthesized the 3-*O*-glucoside (*iso*-syringin) that also matches the RT and fragmentation pattern of the previously unknown peak (Figure 3). Given that *iso*-syringin co-eluted with secoisolariciresinol, which contains a trace amount of m/z 354, quantification of *iso*-syringin suggests that COMT down-regulated switchgrass has at least 43-fold more of the glucoside than the wild-type plants. Therefore, *iso*-sinapyl alcohol and *iso*-syringin were essentially only detected in transgenic plants. To confirm that the presence of *iso*-sinapyl alcohol was not an artifact of the hot water pretreatment, non-pretreated samples were additionally analyzed. Whereas there was no *iso*-sinapyl alcohol detected in the non-pretreated wild-type sample, the transgenic plant had 0.28 μg/ml *iso*-sinapyl alcohol, similar to the 0.29 μg/ml detected in the hot water pretreated sample. Similarly, 3, 4-dimethoxy-5-hydroxycinnamic acid (*iso*-sinapic acid) was also only detected in transgenic plants (0.11 μg/ml). It should be noted that *iso*-sinapyl aldehyde was detectable in transgenic plants (RT 13.06), but not at quantifiable levels. The accumulation of *iso*-sinapyl alcohol is accompanied by the production of its glucoside, *iso*-syringin, which was 11.3-fold higher than syringin in the transgenic plants. The structures of the observed *iso*-sinapyl alcohol related metabolites and potential synthesis routes are shown in Figure 4.

### Quantum chemical calculations of iso-sinapyl radical spin density and reaction thermochemistry

**Figure 5:**
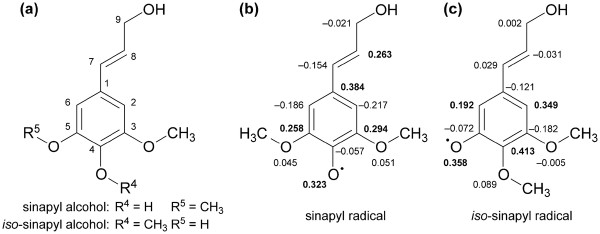
**(a) Chemical structures with atom numbering for sinapyl and *iso*-sinapyl alcohols.** Spin density calculations for (**b**) sinapyl and (**c**) *iso-*sinapyl radicals. Potential coupling sites for *iso*-sinapyl radical are C2, C4, O5 and C6. In comparison to sinapyl radical, *iso*-sinapyl has one less coupling site and lacks reactivity at C8 in particular.

An interesting question regarding the novel monolignol analog is its ability to participate in oxidative couplings with itself. To assess the intrinsic reactivity of *iso*-sinapyl alcohol in relation to other monolignols, quantum chemical calculations were carried out using density functional theory with the ωB97X-D functional. Specifically, electron spin densities were computed to determine the distribution of unpaired spin in the *iso-*sinapyl radical, which indicates relative reactivity at each site, and reaction enthalpies were computed to determine the thermodynamic favorability for various potential radical conjugation reactions. See Additional file 1 for optimized geometries for *iso*-sinapyl alcohol and the *iso*-sinapyl radical, structures and optimized geometries of *iso*-sinapyl homodimers. The electron spin density is defined as the total electron density of electrons of one spin minus the total density of the electrons of the opposite spin. For radical species, sites with the highest spin densities are, in general, expected to be the most reactive [22,23]. The resulting spin density distributions show that, consistent with resonance arguments, the *iso*-sinapyl radical has a lower number of possible sites for conjugation than does the sinapyl radical (Figure 5). For the sinapyl radical, the spin density is highest at C1, followed by O4, C3, C8 and C5. However, reactivity is expected primarily at O4 and C8 because the other positions are sterically hindered by non-hydrogen substituents. In contrast, for the *iso-*sinapyl radical, the unpaired spin resides predominantly on O5, C6, C2 and C4, with the highest spin density localized at C4 (Figure 5). C4 in *iso*-sinapyl alcohol is sterically hindered by a methoxy group and is therefore expected to have low reactivity. Furthermore, C8 is deficient in unpaired spin compared to standard monomer radicals because resonance with the O5 radical site has been abolished. Therefore, the *iso*-sinapyl radical is not expected to undergo coupling at C8.

**Figure 6:**
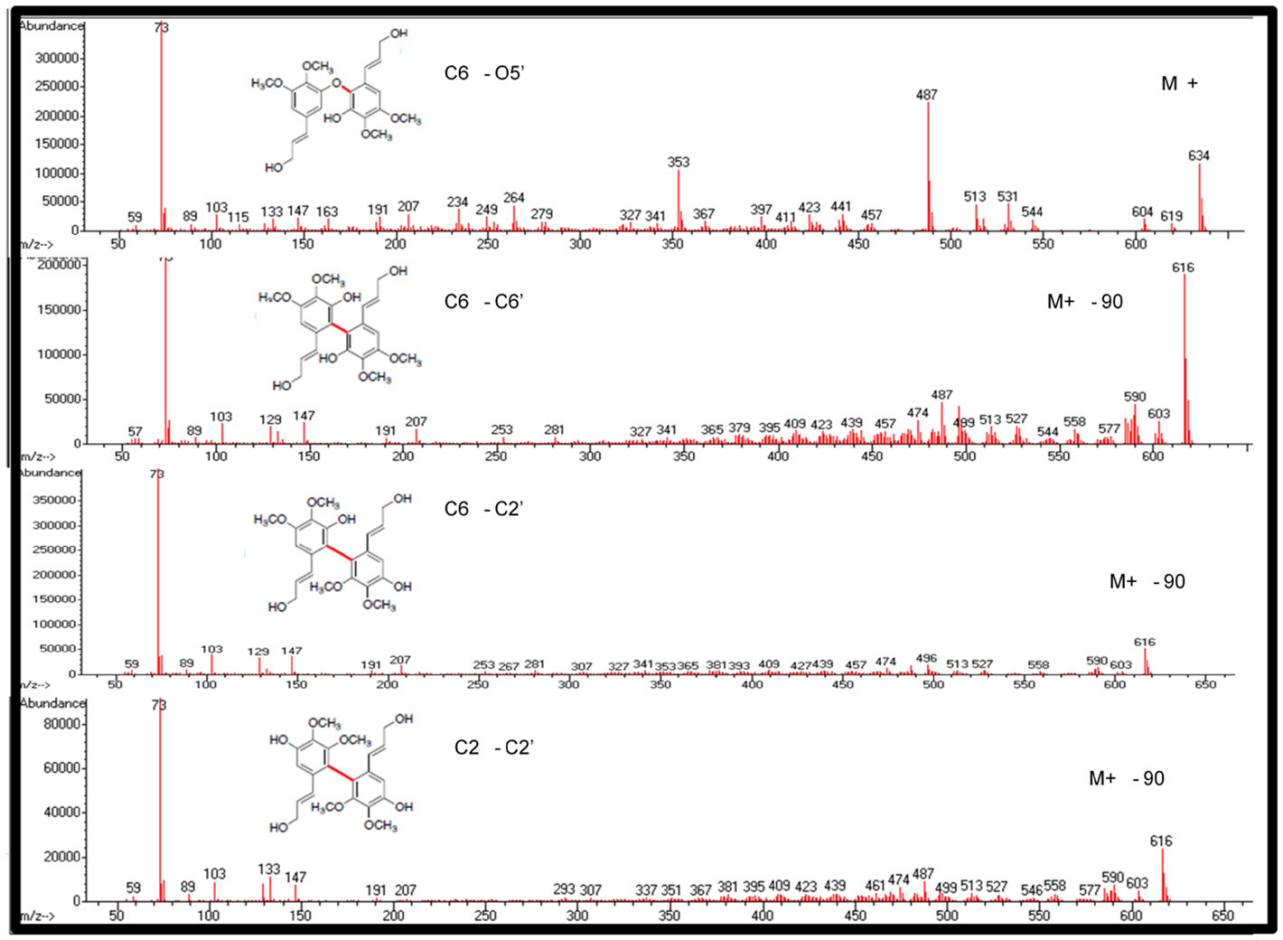
**GCMS EI fragmentation pattern of trimethylsilyl derivatized homodimeric lignans of *iso*-sinapyl alcohol.** “M+” denotes the molecular ion.

It is of interest to consider the expected regioselectivity for reactions of the *iso*-sinapyl radical in the context of lignin polymerization. With highest spin density at O5 and reaction at C8 precluded, reaction at O5 is expected to predominate in couplings of the *iso*-sinapyl radical. The major linkage type in switchgrass lignin is O–8 [24]. In reaction with a conventional lignol radical partner, the directionality of this linkage is enforced to be O(*iso*-sinapyl)–8(partner). Further chain growth would necessarily occur on the partner side, and *iso*-sinapyl moieties would thus occur more frequently at chain termini. In reaction of *iso*-sinapyl radical with a growing chain, reaction via O5 would preclude extension from the *iso*-sinapyl group, and one might expect to see single-unit *iso*-sinapyl appendages on the chain. Continued growth at the penultimate residue would be possible, but might be impeded where preferred extension sites had been consumed in reaction with *iso*-sinapyl radical. Overall, one would expect to find *iso*-sinapyl units relatively enriched at chain termini and as single-unit branches. One might expect a reduced degree of polymerization. However, the GPC analysis indicated that the presence of *iso*-sinapyl alcohol did not affect the molecular weight of synthetic lignins produced by horseradish peroxidase-catalyzed dehydrogenative polymerization of coniferyl alcohol or sinapyl alcohol (Table 2).

**Table 2:** **Horseradish peroxidase-catalyzed dehydrogenative polymerization of sinapyl alcohol (SA) or coniferyl alcohol (CA) with *iso*-sinapyl alcohol (*iso*-SA)**

**Monomer**	**Yield (%)**	${\overset{―}{\text{M}}}_{\text{n}}$**(10**^**2**^**)**	${\overset{―}{\text{M}}}_{\text{w}}/{\overset{―}{\text{M}}}_{\text{n}}$
SA	39.2	6.3	1.3
SA + *iso*-SA	47.7	6.7	1.5
CA	67.1	11.3	1.8
CA + *iso*-SA	62.1	11.1	1.4

### Thermochemistry of iso-sinapyl radical coupling reactions

Based on the spin density calculations and steric considerations, two *iso*-sinapyl radicals could potentially undergo coupling to form C6–O5^′^, C6–C6^′^, C6–C2^′^ and C2–C2^′^ linked lignans. These preferred couplings were determined from reaction enthalpies calculated for the coupling of two *iso*-sinapyl radicals to form homodimers (self-coupling). Each of these self-coupling reactions is strongly exothermic, with the C6–C2^′^ linked lignan computed to have the most favorable reaction enthalpy (−40.9 kcal/mol), followed by C2–C2^′^ (−37.2 kcal/mol), C6–C6^′^ (−35.7 kcal/mol), C2–O5^′^ (−35.2 kcal/mol) and C6–O5^′^ (−31.2 kcal/mol). From *in-vitro* coupling experiments, the C6–C6^′^ and C6–O5^′^ linked lignans were found to be the most abundant, with lower observed production of C6–C2^′^ and C2–C2^′^ linked lignans and no C2–O5^′^ linked lignans detected. Thus, the experimentally observed products are consistent with calculations in that they are all predicted to result from highly exothermic reactions at sites with high spin density.

### Generation of homodimeric lignans of iso-sinapyl alcohol

We hypothesized that the novel monolignol analog and/or its lignan dimers may also be a contributing causal factor to the microbial inhibitory response, given that a number of lignans were observed to be elevated in the hydrolysates of COMT down-regulated switchgrass. To test this hypothesis a number of known lignans were analyzed and a set of dehydrogenation reactions were undertaken with *iso*-sinapyl alcohol to generate homodimeric lignans to test for their presence in the genetically modified biomass. Four homodimeric lignans of *iso*-sinapyl alcohol were readily synthesized by single dehydrogenation reactions (Figure 6). The C6–C6^′^ dimer and the C6–O5^′^ dimer (numbering atoms as in sinapyl alcohol) were the most abundant, whereas there was much less of the C6–C2^′^ dimer and the C2–C2^′^ dimer. These products were expected from the quantum calculations above, but none of these lignans generated from *iso*-sinapyl alcohol has been confirmed in the COMT down-regulated switchgrass.

## Discussion

Plant species have long been known to contain three major monolignols, *p*-coumaryl alcohol, coniferyl alcohol, and sinapyl alcohol [5], and more recently 5-hydroxyconiferyl alcohol has been identified as an additional monolignol that can be incorporated into cell walls, particularly in COMT-deficient poplar (*Populus tremula x alba*) [18]. However, 5-hydroxyconiferyl alcohol remains a relatively minor component of natural lignin, and these results indicated that monolignols other than the three major monolignols can be incorporated into lignin. Although 5-hydroxyconiferyl alcohol was not affected in the present study, its 4-*O*- and 5-*O*-glucosides were greatly accumulated (76-fold and 60-fold, respectively) in COMT down-regulated switchgrass lines. Here, we also demonstrate that the down-regulation of COMT loci in switchgrass additionally resulted in the accumulation of a novel monolignol-like metabolite, *iso*-sinapyl alcohol, its glucoside, *iso*-syringin, and related metabolites *iso*-sinapic acid and *iso*-sinapyl aldehyde. The presence of *iso*-sinapyl alcohol in non-pretreated samples of transgenic plants negates the potential argument that it is an artifact of the mild, hot water pretreatment as a breakdown product of benzodioxane (β-O-5, α-O-5) substructures, which can form from cross-coupling reactions involving radicals of 5-hydroxyconiferyl alcohol. If the latter were the case, *iso*-sinapyl alcohol should be evident in both wild-type and transgenic plants, given that both plant types have at least small quantities of 5-hydroxyconiferyl alcohol. *iso*-Sinapyl alcohol has previously been isolated from the roots of *Ferula sinaica*[25], the leaves of *Croton xalapensis* L. (*Euphorbiaceae*) [26], and the feces of the beetle *Naupactus bipes*[27]. However, it has not been associated with lignin metabolism, it has not been synthesized previously, its biosynthesis in plants has not been investigated, and it has not been previously identified in any of the many previous COMT knockdown studies thus far. It is ironic that blocking of a major methylation step that generates a dimethoxycinnamyl alcohol leads to the appearance of an alternative, novel dimethoxycinnamyl alcohol. A simple explanation for the biosynthesis of *iso*-sinapyl alcohol would be that the reduced activity of COMT allows another methyltransferase to methylate the *para* (4-hydroxyl) position on the aromatic ring of the accumulating sinapyl alcohol precursors. Although this hypothesis lacks experimental support with respect to the presence of such a protein(s) being over-expressed, the accumulations of 5-hydroxyferulic acid, 5-hydroxyconiferaldehyde, and 5-hydroxyconiferyl alcohol glucosides provide metabolite evidence that the global COMT knockdown employed in this study resulted in the accumulation of metabolites that can all be methylated at the *para* position to produce all of the putative *iso*-sinapyl alcohol related precursors observed in this study. Given that native COMT specifically methylates the *meta* (3-hydroxyl and 5-hydroxyl) positions on the phenyl ring of aromatic acids/aldehydes and is precluded from substitution at the *para* position, it is possible that an alternative *para*-specific methyltransferase, similar to *iso*-eugenol 4-*O*-methyltransferase (IEMT; EC 2.1.1.146), described by [28], is able to *para* methylate the accumulating substrates, including the 5-hydroxyferulic acid, 5-hydroxyconiferaldehyde and/or 5-hydroxyconiferyl alcohol, or there may be an alternate pathway that emerges that generates *iso*-sinapic acid and results in the reductive formation of *iso*-sinapyl alcohol. It is generally accepted that COMT acts on 5-hydroxyconiferaldehyde/alcohol as substrates, which explains the reduced level of S-residues in mutants and transgenics with reduced COMT activity. Given that 4-coumarate-CoA ligase EC 6.2.1.12 (4CL) does not display much activity toward sinapic acid in grasses, there would be limited flux of sinapic acid towards sinapyl alcohol in grasses. Thus, *iso*-sinapyl alcohol synthesis from *iso*-sinapic acid was not anticipated, suggesting the aforementioned *para*-methylation of multiple substrates may be the more likely mode of synthesis of the *iso*-sinapyl alcohol related metabolites. Although not readily detected in wild-type plants, it cannot be unequivocally stated that this direct synthesis pathway of *iso*-sinapic acid to *iso*-sinapyl alcohol doesn’t exist in such plants at very low flux and metabolite concentrations. We have detected *iso*-sinapic acid (0.04 μg/ml) in another lignin pathway enzyme (ferulate-5-hydroxylase EC 1.14.-.-; F5H) knockdown line in switchgrass, but *iso*-sinapyl alcohol was not detected in that line. Furthermore, 4-*O*-methylation of monolignol precursors has been postulated in another monocot species, *Vanilla planifolia*, although no enzymatic basis for this conclusion has yet emerged [29]. Additional tracer studies are needed to clarify metabolite flux and the primary pathway leading to the production of *iso*-sinapyl alcohol.

*iso*-Sinapyl alcohol is a monolignol based on its molecular structure, but a key question remains as to whether it is incorporated into the plant cell wall. Quantum chemical calculations demonstrate a reduced number of conjugation sites for *iso*-sinapyl alcohol compared to sinapyl alcohol. The most likely homodimeric lignans formed from single dehydration reactions were predicted to be coupled at C6–C2^′^ (based on atom numbering of sinapyl alcohol), C2–C2^′^, C6–C6^′^, and C6–O5^′^, and these were confirmed by organic synthesis, but none was detected in plant samples. However, two lignan-like metabolites were detected only in COMT down-regulated plants that may be *iso*-sinapyl alcohol-based lignans, but they remain unidentified. A 5-hydroxconiferyl alcohol-coniferyl alcohol heterodimeric benzodioxane structure in the lignin of COMT-deficient *Populus* has been identified [30]*.* An analogous (benzodioxane) metabolite (5-hydroxconiferyl alcohol-sinapyl alcohol), reported by [18,31], may be the lignan RT 15.09 min (molecular ion (M^+^) 620, key m/z 510 420 235), which co-elutes with another lignan that is unique to COMT-deficient plants with key m/z 620 239 354 323 265, the latter three m/z are typical of *iso*-sinapyl alcohol/sinapyl alcohol and suggest the peak may be an *iso*-sinapyl alcohol heterodimeric lignan. However, this has yet to be verified. Another COMT-deficient unique lignan occurred at RT 15.18 min (M^+^ 530 219 354) and is likely an *iso*-sinapyl alcohol-phenolic acid conjugate. The generation of such lignans following pretreatment suggests that *iso*-sinapyl alcohol may be a wall component, but we have not found any evidence to support this. Furthermore, the hypothesis that incorporation of the novel monolignol may result in a lower degree of polymerization of the lignin molecule, was not supported from the GPC analysis. It can be concluded that the presence of *iso*-sinapyl alcohol did not affect the molecular weight of lignin produced by horseradish peroxidase catalyzed dehydrogenative polymerization of either coniferyl alcohol or sinapyl alcohol. In addition, the yield and degree of polymerization (DP_n_) are slightly lower than the literature report [32], in which a larger scale of HRP-catalyzed DHP of sinapyl alcohol in the presence of sodium azide was carried out (0.5 mmol sinapyl alcohol: isolated yield: 54.2%; ${\overset{―}{\text{M}}}_{\text{n}}/{\overset{―}{\text{M}}}_{\text{w}}$: 1.3; DPn : 4.4). Furthermore, a follow-up analysis of the presence of *iso*-sinapyl alcohol in transgenic COMT-deficient switchgrass biomass that had water-soluble constituents removed, followed by sequential enzymatic saccharification with fungal (*Trichoderma reesei*) enzymes, followed then by exposure to cellulolytic microbes *Caldicellulosirupter bescii*, *C. obsidiansis*, and *C. thermocellum*, indicated that no *iso*-sinapyl alcohol was detected in the culture supernatants, whereas sinapyl alcohol, coniferyl alcohol, and 5-hydroxyconiferyl alcohol were present. We conclude that *iso*-sinapyl alcohol is not a major cell wall constituent and should be considered a monolignol analog, given its structure and coupling propensities. This explains the lack of evidence of cell wall structures derived from *iso*-sinapyl alcohol in the present study, whereas we were able to detect benzodioxane substructures by HSQC NMR of internode 1 biomass of switchgrass, as has often been reported in COMT-deficient plants [18,29,32]. Although not detectable in wild-type plants, these substructures constituted 11% of the total lignin linkages, similar to the 12% observed in COMT-deficient *Arabidopsis*[19], and 10% in COMT antisense *Populus*[18]. It should be noted that despite the number of previous studies of various plants species with reduced COMT activity e.g., [15,18,30,33], *iso*-sinapyl alcohol has not been previously identified in such plants, nor has it been identified with the lignin biosynthetic pathway. However, given the associated occurrence of *iso*-sinapic acid, *iso*-sinapyl aldehyde, upstream precursors from the lignin pathway, including 5-hydroxyferulic acid and 5-hydroxyconiferaldehyde, accumulation of glucosides of 5-hydroxyconiferyl alcohol, we conclude that the metabolite flux associated with 5-hydroxyconiferyl alcohol production and subsequent metabolism differs in switchgrass from the other species previously characterized. The accumulation of 5-hydroxyconiferyl alcohol related precursors and glucoside conjugates, provide the substrates that can then be methylated at the *para*- position on the aromatic ring to generate *iso*-sinapyl alcohol and related metabolites. The production of *iso*-sinapyl alcohol and its glucoside, *iso*-syringin, may be non-specific detoxification processes. Other species that have lower rates of production of 5-hydroxyconiferyl alcohol or greater flux of 5-hydroxyconiferyl alcohol into cell walls don’t permit the accumulation of the substrates that would lead to *iso*-sinapyl alcohol production, and hence, a possible explanation for the lack of their detection in previous studies.

The evidence of reduced recalcitrance to deconstruction processes recently reported by [7] may be related to the additional metabolite responses associated the appearance of *iso*-sinapyl alcohol, namely the increased incorporation of phenolic acids of the lignin pathway, particularly ferulic acid, 5-hydroxyferulic acid, and ferulic acid-glycoside conjugates. These changes result in the reduced *p*-coumaric acid to ferulic acid ratio that has been associated with increased forage digestibility in six barley lines [34], but, paradoxically, increased recalcitrance in switchgrass [35]. In monocots, such as corn, the *p*-coumaric acid in secondary cell walls is thought to be bound to lignin, whereas ferulic acid serves as a bridge between lignin and hemicellulose [36]. Sophisticated re-engineering of the cell walls by monolignol substitution with methyl caffeic acid, caffeoylquinic acid, and feruloylquinic acid, has succeeded in creating cell walls that have less lignin and are more easily deconstructed [37]. The COMT-deficient switchgrass was reported to have an increased dry matter digestibility [7]. Similar responses were reported for COMT-deficient tall fescue [6,15]. Although increased incorporation of ferulic acid into cell walls may lower cell wall recalcitrance, ferulic acid is thought to be one of the most inhibitory factors contributing to the biodegradability of biomass [34]. Phenolic acids and aldehydes derived from cell wall biodegradation are known fermentation inhibitors [38-40]. These cumulative responses are likely correlated with the metabolic block in the lignin pathway plus the observed lowered recalcitrance, yielding increased enzymatic sugar release from cell walls during deconstruction. Therefore, the sum of the increase of many phenolic constituents in COMT-deficient plants, including ferulic acid, its many conjugates, and the phenolic aldehydes, may explain, at least in part, the observed increase in the inhibitory nature of these plants relative to wild-type controls. When added separately to media, *iso*-sinapyl alcohol (up to 50 μg/ml) and *iso*-sinapic acid (up to 25 μg/ml) were not inhibitory to the growth of *C. thermocellum* cultures (data not shown). The complex changes in the cell walls of transgenic biomass that include the greater release of phenolic acids and aldehydes must be tolerated by cellulolytic microbes. However, given the significant increase in the mass yield of fermentation products with the COMT transgenic switchgrass and the observation that simple washing allows efficient fermentation by yeast and *C. thermocellum*[7], these transgenic biomass sources remain valuable and viable future resources for biofuels.

## Conclusions

Down-regulation of the COMT activity in the lignin biosynthetic pathway of switchgrass resulted in the expected reduction in sinapyl alcohol and related metabolites, but increased phenolic acids of the lignin pathway, particularly ferulic acid, 5-hydroxyferulic acid, and ferulic acid-glycoside conjugates, and related phenolic aldehydes, including vanillin and 5-hydroxyconiferaldehyde. The accumulation of these lignin pathway related phenolic acids and aldehydes explain, in part, the observed increase in the inhibitory nature of the transgenic biomass relative to wild-type controls, following direct fermentation (with no water washing of biomass) with *C. thermocellum*. Additionally, down-regulation of the COMT activity revealed the presence of a novel monolignol-like metabolite, identified as *iso*-sinapyl alcohol and related metabolites of *iso*-sinapic acid, *iso*-sinapyl aldehyde, and *iso*-syringin in both non-pretreated, as well as hot water pretreated transgenic biomass. The metabolomic results suggest the increased activity of a *para*-methyltransferase on accumulating substrates related to 5-hydroxyconiferyl alcohol, concomitant with the reduced COMT activity, likely generates the *iso*-sinapyl alcohol related metabolites. *iso*-Sinapyl alcohol is considered a monolignol analog given that there was no evidence that it was integrated in cell walls, including the absence of homodimeric lignans of *iso*-sinapyl alcohol in the transgenic biomass, and no observable effect of *iso*-sinapyl alcohol on the dehydrogenative polymerization of monolignols. The emergence of a previously unknown pathway following transgenesis highlights the need to fully characterize the metabolic consequences of transgenesis by metabolomic analyses, and demonstrates transgenic biomass may have varied biological properties that require assessment.

## Methods

### Plant materials and hydrolysate preparation

Samples of the T1 COMT transgenic switchgrass variety Alamo and corresponding T1 wild-type were received from the Samuel Roberts Noble Foundation and have been described previously [7]. Switchgrass samples were milled in a Wiley mill through a 0.8 mm screen. Pretreatment was conducted using the tubular batch method from [41], except only one sand bath (Omega FSB1, Techne Co., Princeton, NJ) was used to heat the 4 × 0.5 inch pretreatment tubes. Biomass was soaked in nine fold excess deionized water overnight (~18 h) and centrifuged at 11000 g for 5 min in 50 ml disposable centrifuge tubes (Falcon) in a Sorvall Legend XTR (Thermo Scientific, Waltham, MA) centrifuge. The dry biomass solids were loaded in the pretreatment tubes, each of which holds approximately 2.5 g, and the tubes were heated in boiling water for 2 minutes prior to heating in the sand bath at 180°C for 25 min., followed by ice bath quenching. Treated biomass from each tube was used directly after the water content was determined. Anaerobic *C. thermocellum* fermentations were conducted in 120 ml serum vials containing 60 ml of MTC medium [42], and one gram hot water pretreated switchgrass at 58°C shaking at 150 rpm. Fermentations continued for 337 h, but were essentially complete by 200 h based upon weight loss analysis [7]. Fermentation biomass composition and fermentation products were analyzed by HPLC, as described previously [42].

### Metabolite profiling of hydrolysates

250 μl of thawed hydrolysate and 15 μl of sorbitol (0.1000 g/100 ml aqueous) were transferred to a vial and concentrated to dryness under a stream of N_2_. The internal standard was added to correct for subsequent differences in derivatization efficiency and changes in sample volume during heating. Dried extracts were dissolved in 500 μl of silylation–grade acetonitrile followed by the addition of 500 μl *N*-methyl-*N*-trimethylsilyltrifluoroacetamide (MSTFA) with 1% trimethylchlorosilane (TMCS) (Thermo Scientific, Bellefonte, PA), and samples then heated for 1 h at 70°C to generate trimethylsilyl (TMS) derivatives [43]. After 1 day, 1-μl aliquots were injected into an Agilent Technologies Inc. (Santa Clara, CA) 5975C inert XL gas chromatograph-mass spectrometer, fitted with an Rtx-5MS with Integra-guard (5% diphenyl/95% dimethyl polysiloxane) 30 m × 250 μm × 0.25 μm film thickness capillary column. The standard quadrupole GCMS was operated in the electron ionization (EI) (70 eV) mode, with 6 full-spectrum (50–650 Da) scans per second. Gas (helium) flow was 1.33 ml per minute with the injection port configured in the splitless mode. The injection port, MS Source, and MS Quad temperatures were 250°C, 230°C, and 150°C, respectively. The initial oven temperature was held at 50°C for 2 min and was programmed to increase at 20°C per min to 325°C and held for another 11 min, before cycling back to the initial conditions. A large user-created database (>1600 spectra) of mass spectral EI fragmentation patterns of TMS-derivatized compounds, as well as the Wiley Registry 8th Edition combined with NIST 05 mass spectral database, were used to identify the metabolites of interest to be quantified. Peaks were reintegrated and reanalyzed using a key selected ion, characteristic m/z fragment, rather than the total ion chromatogram, to minimize integrating co-eluting metabolites. The extracted peaks of known metabolites were scaled back up to the total ion current using predetermined scaling factors. Unidentified metabolites used the scaling factor for the internal standard (sorbitol) and were denoted by their RT as well as key m/z fragments. The mass-to-charge ratios used as extracted ions were as follows: *iso*-sinapyl alcohol (354), *iso*-sinapic acid (368), *iso*-syringin (354), 5-hydroxyconiferyl alcohol-4-*O*-glucoside (412), 5-hydroxyconiferyl alcohol-4-*O*-glucoside (412), 3,4-dihydroxybenzoic acid (370), xanthine (368), hypoxanthine (265), succinic acid (247), guanosine (324), uracil (241), citraconic acid (259), guanine (352), 5-hydroxyferulic acid (411), uridine (258), maleic acid (245), secoisolariciresinol (560), 5-*oxo*-proline (156), adenine (264), 1-*O-trans-*feruloylglycerol (249), vanillin (297, 194), ferulic acid (338), adenosine (236), *p*-coumaric acid (308), caffeic acid (396), *p*-hydroxybenzaldehyde (392, 194), coniferyl alcohol (324), 5-hydroxyconiferyl alcohol (412), coniferyl aldehyde (323), guaiacylglycerol (297), sinapyl aldehyde (353), syringylglycerol (327), *p*-hydroxyphenylpyruvic acid (396), syringaresinol (327), pinoresinol (502), hydroxymethylfurfural (183). Peaks were quantified by area integration and the concentrations were normalized to the quantity of the internal standard recovered, volume of sample extracted, derivatized, and injected.

### Statistical analyses

Three replicate samples were analyzed per plant line. There were five wild-type lines and four COMT-deficient lines that were analyzed. Plant line was considered the experimental unit. The metabolite data were averaged by construct (COMT-deficient versus wild-type). Construct differences were analyzed by Student’s t-tests with differences considered significant at P ≤ 0.05.

### Metabolite synthesis

#### Ethyl trans-3,4-dimethoxy-5-hydroxycinnamate

To 3, 4-dimethoxy-5-hydroxybenzaldehyde (211.6 mg, 1.16 mmol) and 487.6 mg (1.40 mmol, 1.2 equiv) of carbethoxymethylene triphenylphosphorane in a 5-ml round-bottom flask containing a magnetic stir bar was added 2.8 ml of reagent-grade toluene. The mixture was stirred and placed in an oil bath at 80°C for 30 min. After the now-homogeneous solution had cooled to room temperature, it was loaded directly onto a 10 × 120 mm column of silica gel packed in 2:1 hexanes:ethyl acetate, and the product was eluted with the same solvent mixture. Product-containing fractions, identified by thin-layer chromatography analysis with visualization by UV-shadowing and staining with phosphomolybdic acid (10% in ethanol), were combined and evaporated to dryness. The crude product (ca. 275 mg) was recrystallized from 10 volumes (i.e., 2.75 ml) of hexanes plus sufficient chloroform (ca 1.1 ml) to dissolve the product in boiling solvent. After removal of the mother liquor with a Pasteur pipet pulled to a capillary tip, the crystals were washed with 2 × 1 ml of ice-cold 3:1 hexanes:chloroform and dried *in vacuo* to afford 210.2 mg (68%) of the product. ^1^ H NMR (400 MHz, CDCl_3_) δ 7.56 (d, *J* = 16 Hz, 1 H), 6.81 (d, *J* = 2.0 Hz, 1 H), 6.64 (d, *J* = 2.0 Hz, 1 H), 6.32 (d, *J* = 16 Hz, 1 H), 5.89 (s, 1 H), 4.26 (q, *J* = 7.2 Hz, 2 H), 3.93 (s, 3 H), 3.89 (s, 3 H), 1.34 (t, *J* = 7.2 Hz, 3 H); ^13^C NMR (100 MHz, CDCl_3_) δ 167.0, 152.4, 149.4, 144.4, 137.3, 130.4, 117.7, 108.0, 104.0, 61.0, 60.5, 55.9, 14.3.

#### trans-3,4-Dimethoxy-5-hydroxycinnamyl alcohol (iso-sinapyl alcohol)

Ethyl (*E*)-3, 4-dimethoxy-5-hydroxycinnamate (132.8 mg, 0.50 mmol) was placed in a 10-ml round-bottom flask and dried azeotropically by two cycles of dissolution in toluene (ca. 2 ml), followed by rotary evaporation. After a stir bar was added, the flask was fitted with a rubber septum, evacuated, heated to 40°C for 20 min, and then filled with dry nitrogen. Anhydrous toluene (2.8 ml) was added, the stirred suspension was cooled to 0°C, and DIBAL (1.7 ml of a 1.0 M solution in toluene, 3.4 equiv) was added dropwise over 10 minutes. After 1 h, TLC indicated that the starting material had been consumed. The reaction was quenched by the addition of 0.5 ml of ethanol at 0°C, then partitioned between water saturated with potassium bitartrate (10 ml) and ethyl acetate (15 ml). The aqueous layer was further extracted with 3 × 15 ml of ethyl acetate, and the combined organic layers were dried over sodium sulfate and filtered through Celite. After the solvent was evaporated, the crude product was purified by chromatography on a 10 × 150 mm column of silica gel using 1:3 hexanes:ethyl acetate to afford the product in > 95% yield. ^1^ H NMR (400 MHz, CDCl_3_) δ 6.66 (d, J = 1.9 Hz, 1 H), 6.52 (d, J = 2.0 Hz, 1 H), 6.49 (dt, J = 16 Hz, 1.5 Hz, 1 H), 6.26 (dt, J = 16 Hz, 5.8 Hz, 1 H), 5.88 (br s, 1 H), 4.31 (dd, J = 5.8 Hz, 1.4 Hz, 2 H), 3.89 (s, 3 H), 3.87 (s, 3 H), 1.69 (br s, 1 H); ^13^C NMR (100 MHz, CDCl_3_) δ 152.4, 149.2, 135.3, 132.8, 130.7, 128.0, 106.4, 102.3, 63.4, 60.0, 55.7. ^1^ H NMR data matched those previously reported [25].

#### iso-Syringin

*iso*-Sinapyl alcohol (5.6 mg) and acetobromo-α-d-glucose (11.3 mg) were dissolved in anhydrous methanol and allowed to stir under an inert atmosphere in a reacti-vial. Sodium methoxide (0.5 M in methanol) was slowly added dropwise by syringe until the solution was pH 9.5-10. The reaction was monitored by TLC and the pH was checked after several hours. Additional sodium methoxide was added to maintain pH. The reaction was allowed to stir for 18 hours at room temperature and an aliquot was removed, evaporated, TMS-derivatized, and analyzed by GCMS, as outlined above.

#### Lignan generation

Oxidation of *iso*-sinapyl alcohol was performed with silver carbonate, essentially as described by [18]. The monolignol was dissolved at 0.1 M in 2:1 benzene:acetone. Small portions (1.5–6 mg, 5–20 mmol) of finely pulverized Ag_2_CO_3_ were distributed into reacti-vials and then weighed accurately. Appropriate volumes of monolignol solution were added to produce stoichiometries of 1:1 or 2:1 Ag^+^:monolignol, and the mixtures were stirred overnight at room temperature. Each reaction mixture was applied to a small column of silica gel (2.5-cm bed in a Pasteur pipet), washed through with ethyl acetate, and evaporated to dryness. GCMS analysis was performed after silylation as described under *Metabolite profiling of hydrolysates*.

### Quantum chemical computational methods

To identify low-energy conformers of *iso*-sinapyl alcohol and lignans, conformational scans were performed using the MM3 force field [44], as implemented in the Tinker suite of programs [45]. The ten lowest-energy conformers for each species were then optimized at the B3LYP/6-31 + G(d,p) level of theory [46,47] using the program NWChem [48]. For *iso*-sinapyl radical, the O5 hydrogen was removed from the corresponding alcohol conformers and the structures were re-optimized using B3LYP. The single lowest-energy conformer for each species was then re-optimized using the ωB97X-D range-separated hybrid density functional with empirical dispersion corrections [49] as implemented in the program Gaussian09 [50] with the 6-31 + G(d,p) basis set. Vibrational frequencies were computed to confirm that all optimized structures were true minima. Electron spin densities based on Mulliken population analyses were used to quantify the degree of unpaired spin at various sites in the radicals, and reaction enthalpies were computed to assess the favorability of various radical conjugations. For the reaction enthalpies, corrections for basis set superposition error were included using the counterpoise method [51]. All energies were calculated for the gas phase.

### Determination of the effect of iso-sinapyl alcohol on dehydrogenative polymerization

The horseradish peroxidase (HRP) catalyzed dehydrogenative polymerizations (DHP) of sinapyl alcohol (SA), or combination of sinapyl alcohol and *iso*-sinapyl alcohol (*iso*-SA) were carried out in the presence of sodium azide, according to [52]. In addition, HRP-catalyzed dehydrogenative polymerizations of coniferyl alcohol (CA) or a combination of CA and *iso*-SA, were carried out in the absence of sodium azide, according to the so-called bulk polymerization method [53]. Isolated DHP product was then dissolved in THF (1 mg/ml), filtered through a 0.45 μm filter and placed in a 2 ml auto-sampler vial. The molecular weight distributions of the DHP products were then analyzed on an Agilent GPC SECurity 1200 system equipped with four Waters Styragel columns (HR1, HR2, HR4, HR6), Agilent refractive index detector and Agilent UV detector (270 nm), using THF as the mobile phase (1.0 ml/min) with injection volumes of 20 μl. A calibration curve was constructed based on eight narrow polystyrene standards ranging in molecular weight from 1.5 × 10^3^ to 3.6 × 10^6^ g/mol. Data collection and processing were performed using Polymer Standards Service WinGPC Unity software (Build 6807). Molecular weights (M_n_ / M_w_) were calculated by the software relative to the universal polystyrene calibration curve.

HRP-catalyzed dehydrogenative polymerization of sinapyl alcohol (SA): Two solutions were prepared for the polymerization. Solution A consisted of 10.5 mg (0.05 mmol) of SA and 1.0 mg of HRP (100 U mg^−1^, Fluka) dissolved in 10 ml of distilled water; solution B consisted of 3.3 mg (0.05 mmol) of sodium azide dissolved in 10 ml of 0.02% hydrogen peroxide (0.6 mmol). Solutions A and B were gradually added to 5 ml of sodium phosphate buffer (0.1 M, pH 6.5) over 30 min at 25°C and allowed to stand for 24 h. The precipitates of the resulting DHP were collected by centrifugation and washed with distilled water and dried by vacuum oven (4.9 mg). HRP-catalyzed dehydrogenative polymerization of 4:1 SA and *iso*-SA: Two solutions were prepared for the polymerization. Solution A consisted of SA (8.4 mg, 0.04 mmol) and *iso*-SA (2.1 mg, 0.01 mmol) and 1.0 mg of HRP, and solution B were prepared as above. Solutions A and B were gradually added as above, and the precipitates of the resulting DHP were collected as above (5.1 mg). HRP-catalyzed dehydrogenative polymerization of CA: Two solutions were prepared for the polymerization. Solution A consisted of CA (8.5 mg, 0.05 mmol) and 1.0 mg of HRP, and solution B were prepared as above. Solutions A and B were gradually added as above, and the precipitates of the resulting DHP were collected as above (5.7 mg). HRP-catalyzed dehydrogenative polymerization of 4:1 CA and *iso*-SA: Two solutions were prepared for the polymerization. Solution A consisted of CA (6.8 mg, 0.04 mmol) and *iso*-SA (2.1 mg, 0.01 mmol) and 1.0 mg of HRP, and solution B were prepared as above. Solutions A and B were gradually added and the precipitates of the resulting DHP were collected, as above (4.2 mg).

## Abbreviations

COMT: Caffeic acid 3-*O*-methyltransferase EC 2.1.1.68; GCMS: Gas chromatography–mass spectrometry; H: Hydroxyphenyl; G: Guaiacyl; S: Syringyl; RT: Retention time; DIBAL: Diisobutylaluminum hydride; IEMT: *iso*-eugenol 4-*O*-methyltransferase EC 2.1.1.146; 4CL: 4-coumarate-CoA ligase EC 6.2.1.12; F5H: Ferulate-5-hydroxylase EC 1.14.-.-; M^+^: Molecular ion; m/z: Mass-to-charge ratio; EI: Electron ionization; TMS: Trimethylsilyl; MSTFA: *N*-methyl-*N*-trimethylsilyltrifluoroacetamide; TMCS: Trimethylchlorosilane; SA: Sinapyl alcohol; *iso*-SA: *iso*-sinapyl alcohol; CA: Coniferyl alcohol; HRP: Horseradish peroxidase; DHP: Dehydrogenative polymerization; THF: Tetrahydrofuran; ${\overset{―}{\text{M}}}_{\text{n}}$: Number average molecular weight; ${\overset{―}{\text{M}}}_{\text{w}}$: Weight average molecular weight.

## Competing interests

The authors declare that there are no competing financial or nonfinancial interests.

## Authors’ contributions

TJT participated in the design of the study, carried out the experimental work, the analysis and interpretation of data and drafted the manuscript. RFS participated in the design of the study, conducted the experimental work, contributed to the writing and editing of the manuscript. NLE participated in the design of the study and conducted the experimental work. MZM participated in data analysis and interpretation. AKS and JMP participated in the design of the study, carried out the experimental work, the data analysis, and contributed to the writing of the manuscript. JCM contributed to the writing and editing of the manuscript. RS, NJ and YP carried out the experimental work, the analysis and interpretation of data. AJR participated in the design of the study, the analysis and interpretation of data, and contributed to the writing of the manuscript. CYH carried out the experimental work and data analysis. CF and Z-YW participated in the design of the study, carried out the experimental work, and edited the manuscript. BHD participated in the design of the study and edited the manuscript. RAD and JRM conceived and participated in the design of the study, and contributed to the writing and critical revision of the manuscript. All authors read and approved the final manuscript.

## Supplementary Material

Additional file 1 **Optimized geometries for *iso*-sinapyl alcohol and *iso*-sinapyl radical.** Click here for file
