# Supplementary material for: Down-regulation of the caffeic acid O-methyltransferase gene in switchgrass reveals a novel monolignol analog
Source: Biotechnol Biofuels. 2012 Sep 21;5:71. doi: 10.1186/1754-6834-5-71 (PMC3524654; doi:10.1186/1754-6834-5-71)
Supplement: Additional file 1 — Optimized geometries for iso-sinapyl alcohol and iso-sinapyl radical. [file 1754-6834-5-71-S1.pdf]

Additional file 1

Optimized geometries for *iso*-sinapyl alcohol and *iso*-sinapyl radical

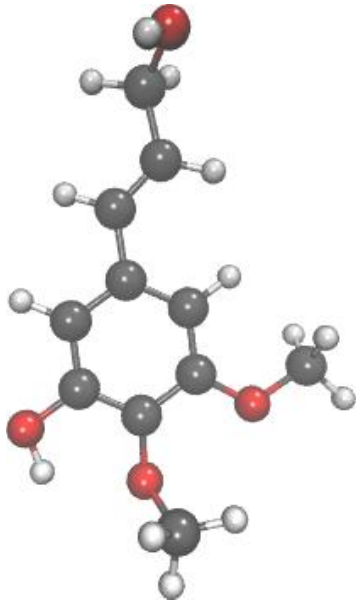

***iso*-sinapyl alcohol**

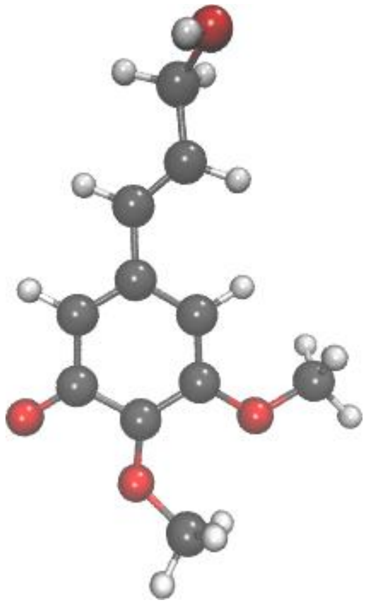

***iso*-sinapyl radical**

In the lowest-energy conformer of *iso*-sinapyl alcohol and *iso*-sinapyl radical, the γ-hydroxyl group is oriented such that the hydrogen is pointed back over the vinyl π cloud as previously reported for *p*-coumaryl, coniferyl and sinapyl alcohols (Rodrigo, 2011). Whereas the methoxy groups in the three traditional monolignols were found previously to be oriented in the plane of the aromatic ring, in the current work the 4-methoxy group in *iso*-sinapyl alcohol is out-of-plane and is oriented in the same direction as the γ-hydroxyl group.

Reference:

Rodrigo CP, James III WH, Zwier TS: **Single-conformation ultraviolet and infrared spectra of jet-cooled monolignols: *p*-coumaryl alcohol, coniferyl alcohol, and sinapyl alcohol.** *J. Am. Chem. Soc*. 2011, **133**:2632–2641.

Structures and optimized geometries of *iso*-sinapyl homodimers

Orientation	Initial Conjugation Product (modeled)	Final Tautomerized Products
C6–O5′	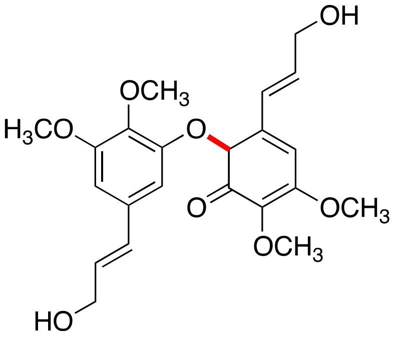	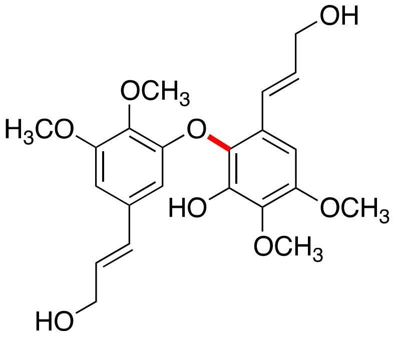
C6–C6′	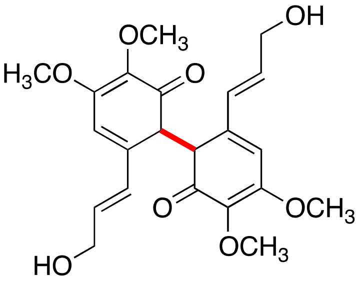	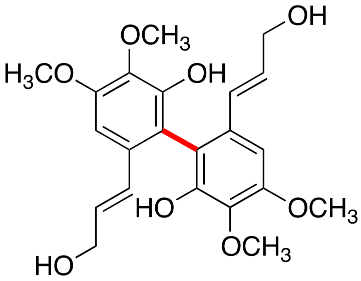
C6–C2′	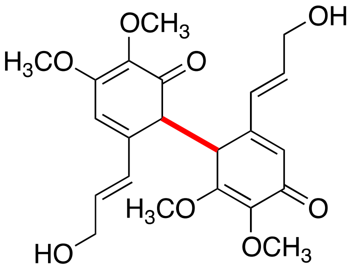	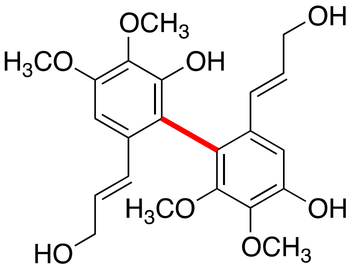
C2–C2′	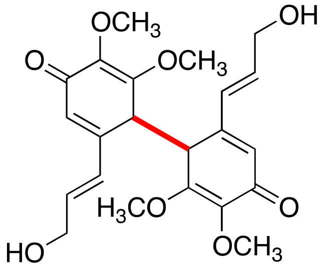	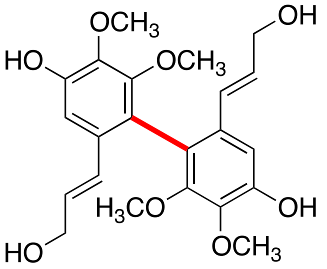
C2–O5′	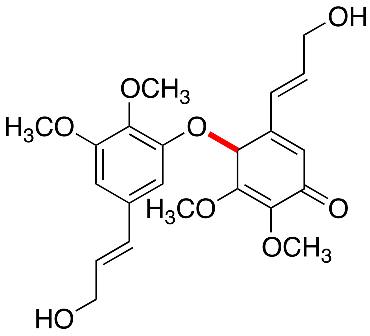	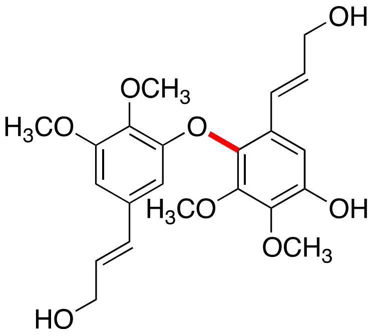

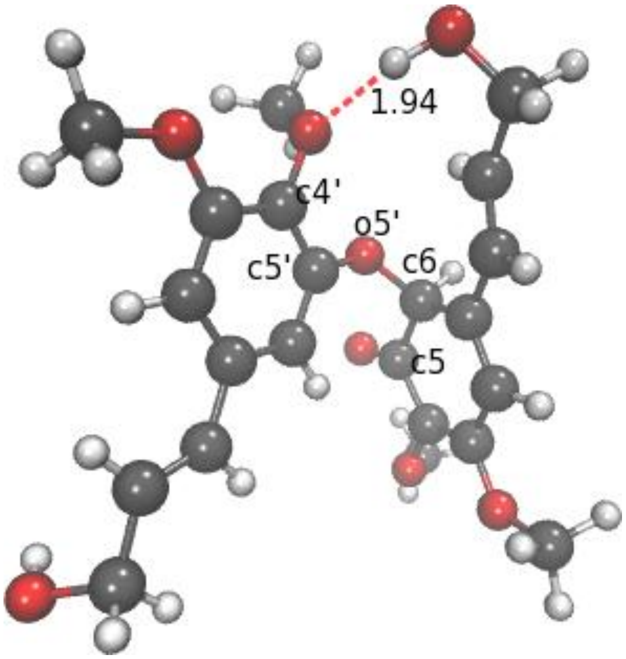

C6–O5′

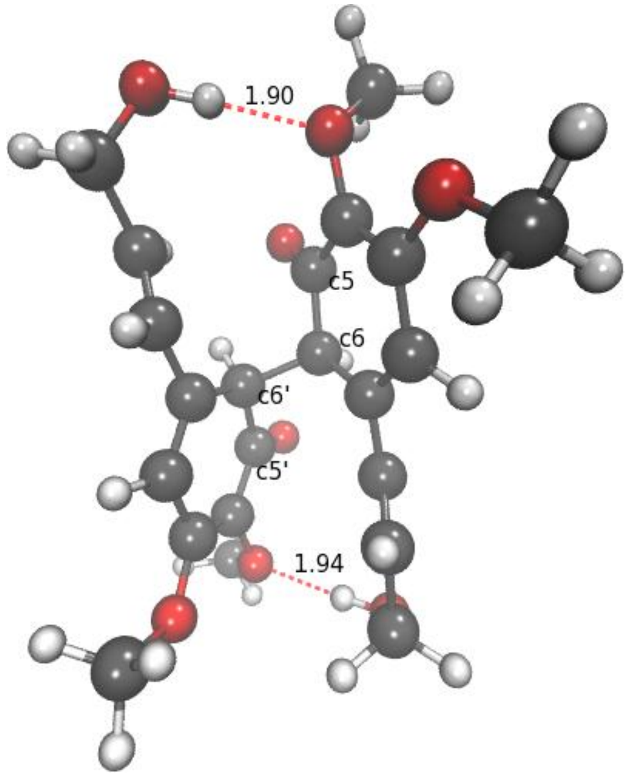

C6–C6′

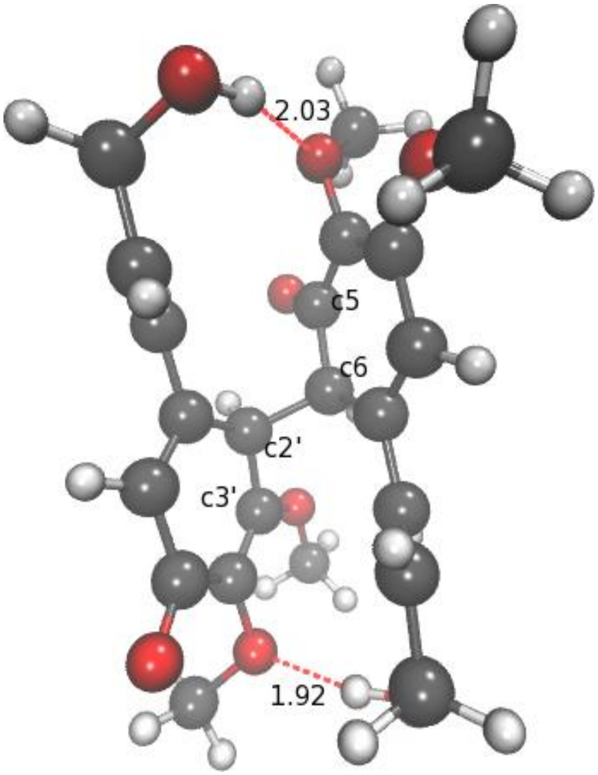

C6–C2′

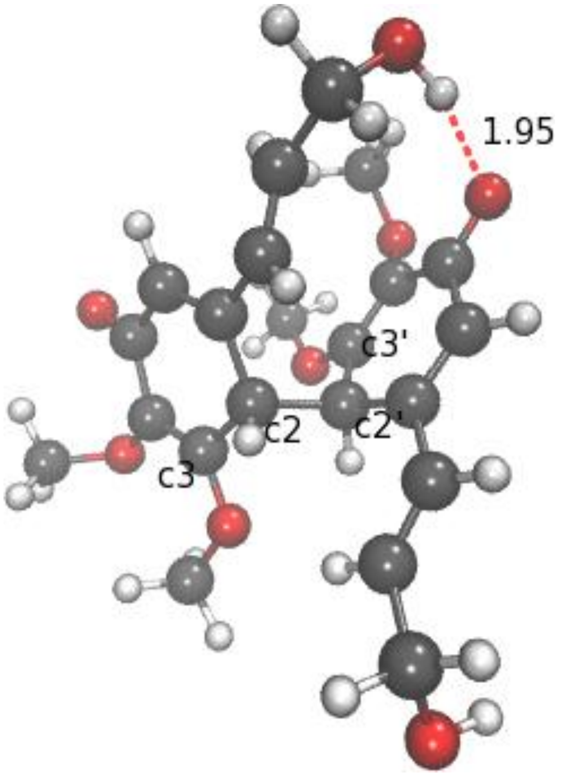

C2–C2′

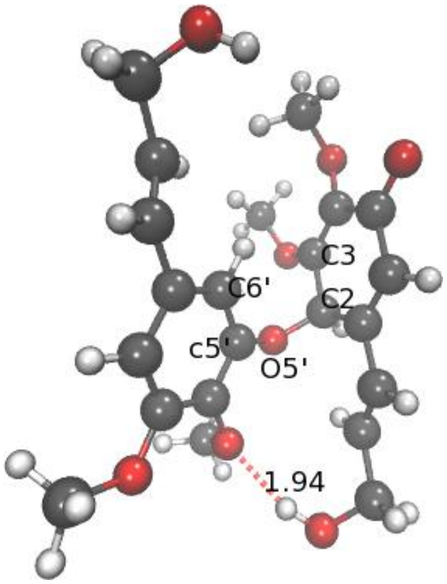

C2–O5′

C6–**O5′**: Both the 4-methoxy and γ-hydroxyl groups are oriented out-of-plane with respect to the aromatic ring in both *iso*-sinapyl units. The γ-hydroxyl hydrogen of one *iso*-sinapyl subunit is hydrogen bonded to the 4-methoxy group of the other subunit. The C5–C6–O5′–C5′ and C6–O5′–C5′–C4′ dihedrals are 81.8 and 143 degrees, respectively.

C6–**C6′:** Both of the *iso*-sinapyl subunits is hydrogen bonded between the γ-hydroxyl hydrogen of one subunit and the 4-methoxy group of the other subunit. The *R*,*R* stereoisomer was found to be more stable than *R,S*. For the *R,R* isomer, the C5–C6–C6′–C5′ dihedral is 152.2 degrees.

C6–C2′: This homodimer also contains two intramolecular hydrogen bonds similar to C6–C6. The *R*,*R* stereoisomer was computed to be energetically more stable than *R*,*S*. The C5–C6–C2′–C3′ dihedral angle in the *R*,*R* stereoisomer is 156.9 degrees.

C2–C2′: The two *iso*-sinapyl subunits are hydrogen bonded via the γ-hydroxyl of one subunit and O5 of the other subunit. The *R*,*S* stereoisomer was found to be energetically more stable than *R*,*R*. The C3–C2–C2′–C3′ dihedral angle in the *R*,*S* isomer is 83.9 degrees.

C2–**O5′**: An intramolecular hydrogen bond exists between the γ-hydroxyl hydrogen of one *iso*-sinapyl unit and the 4-methoxy group of the other *iso*-sinapyl unit. The C6′–C5′–C5′–C2 and C5′–O5′–C2–C3 are 45.37 and -84.91 respectively.

Cartesian coordinates of the lowest-energy conformer of *iso*-sinapyl alcohol, *iso*-sinapyl radical and four homodimers optimized at the ωB97X-D/6-31+G(d,p) level of theory:

iso-sinapyl alcohol

```
C  -0.830869  -0.592224  -0.254147
C  -0.231843   0.678277  -0.253951
C   1.153294   0.802294  -0.192559
C   1.952548  -0.349528  -0.119533
C   1.354208  -1.607621  -0.145663
C  -0.031628  -1.733666  -0.212133
C  -2.293941  -0.767417  -0.302708
C  -3.230910   0.174691  -0.148733
C  -4.702703  -0.105131  -0.217730
O  -5.383260   0.302233   0.961435
O   1.825318   1.981648  -0.204791
C   1.078664   3.176701  -0.316470
O   3.324499  -0.318654  -0.105648
C   3.911887   0.231386   1.073706
O   2.129154  -2.717347  -0.109562
H  -4.974876  -0.140333   1.711650
H   3.049494  -2.420034  -0.137745
H  -0.852337   1.562141  -0.324858
H  -0.468375  -2.726634  -0.225300
H  -2.624421  -1.791967  -0.477194
H  -2.964230   1.209920   0.058920
H  -5.165789   0.470574  -1.025069
H  -4.876913  -1.170513  -0.427005
H   0.395502   3.302074   0.532082
H   1.810198   3.984285  -0.315611
H   0.509864   3.202797  -1.253331
H   4.988315   0.090633   0.969512
H   3.682939   1.296403   1.158583
H   3.552714  -0.298965   1.963422
```

iso-sinapyl radical

```
C  -0.084922  -1.828584  -0.151704
C  -0.835976  -0.686614  -0.200529
C  -0.176859   0.582862  -0.188764
C   1.198926   0.697170  -0.105761
C   1.998083  -0.468432  -0.007788
C   1.358784  -1.791641  -0.051597
C  -2.302594  -0.788107  -0.275596
C  -3.193012   0.207137  -0.195097
```

C	-4.676316	-0.003380	-0.277522
O	-5.352885	0.507528	0.861500
O	1.861517	1.886097	-0.146946
C	1.108501	3.076158	-0.261385
O	3.321003	-0.514972	0.077227
C	4.119407	0.558337	0.574604
O	2.043312	-2.827113	0.011323
H	-5.002659	0.069512	1.643208
H	-0.774574	1.480343	-0.279767
H	-0.539732	-2.813609	-0.175359
H	-2.678925	-1.803158	-0.404333
H	-2.881808	1.239036	-0.040049
H	-5.093936	0.546397	-1.126446
H	-4.899703	-1.069016	-0.429741
H	0.428468	3.202738	0.589644
H	1.836518	3.886815	-0.265923
H	0.536886	3.097123	-1.197046
H	5.078820	0.099273	0.812058
H	4.247053	1.335167	-0.179867
H	3.675876	0.987628	1.475620

C6–O5′ dimer

C	0.800990	1.977196	-0.119498
C	2.196171	1.977794	0.017423
C	2.922948	0.818637	-0.236603
C	2.259845	-0.359452	-0.622516
C	0.874328	-0.350308	-0.779360
C	0.149161	0.814156	-0.527048
C	-0.022135	3.172855	0.138756
C	0.388918	4.365736	0.582634
C	-0.535903	5.527183	0.797453
O	-0.179344	6.646783	-0.002042
O	4.268160	0.712243	-0.127455
C	4.996423	1.852233	0.280743
O	2.975259	-1.514146	-0.770769
C	3.297749	-1.862730	-2.116610
O	0.310664	-1.521948	-1.200086
H	-0.183394	6.370205	-0.923540
H	2.706642	2.883647	0.317624
H	-0.927004	0.829374	-0.654767
H	-1.087060	3.039698	-0.055681
H	1.439895	4.563512	0.786804
H	-1.576366	5.224026	0.612004
H	-0.471690	5.885140	1.829514

H	4.885876	2.673842	-0.437435
H	4.684698	2.189627	1.276607
H	6.040151	1.541210	0.316340
H	2.385757	-1.969931	-2.711755
H	3.952138	-1.102742	-2.556299
H	3.822187	-2.817481	-2.064649
C	-0.975050	-1.834170	0.847098
C	-2.057800	-1.333396	1.474835
C	-3.199856	-0.782837	0.761690
C	-3.246602	-0.724462	-0.606468
C	-2.101593	-1.159834	-1.396723
C	-0.949160	-1.882126	-0.662610
C	0.154205	-2.377235	1.601944
C	1.100864	-3.184036	1.105554
C	2.276426	-3.690382	1.886814
O	3.480063	-3.568878	1.158326
O	-4.260917	-0.290072	1.421806
C	-4.253729	-0.199344	2.837006
O	-4.273869	-0.073907	-1.209048
C	-5.015196	-0.832130	-2.161613
O	-2.022697	-0.999895	-2.604243
H	3.452645	-2.744332	0.648361
H	-2.084373	-1.352626	2.558384
H	-1.074854	-2.935272	-0.952595
H	0.201887	-2.086782	2.651371
H	1.083595	-3.476200	0.058351
H	2.338728	-3.167603	2.853402
H	2.153001	-4.759289	2.098619
H	-4.207711	-1.188473	3.305213
H	-3.427896	0.424725	3.194602
H	-5.199208	0.274782	3.097430
H	-5.813071	-0.175175	-2.508912
H	-4.384952	-1.128660	-3.003742
H	-5.454173	-1.716760	-1.684581

C6–C6′ dimer

C	-0.125537	1.359776	-0.594512
C	-1.074263	2.090075	0.024277
C	-2.466027	1.667292	0.065968
C	-2.896187	0.561929	-0.611813
C	-1.967772	-0.263595	-1.361740
C	-0.478667	0.067999	-1.287201
C	1.278289	1.785515	-0.662132
C	1.968697	2.311894	0.352465

C	3.443285	2.617423	0.293639
O	4.062541	2.269759	-0.914463
O	-3.388720	2.357016	0.755673
C	-3.013325	3.495652	1.514315
O	-4.193037	0.145194	-0.488607
C	-5.000294	0.308818	-1.656805
O	-2.336726	-1.232241	-2.017227
H	4.160151	1.304374	-0.958472
H	-0.802006	3.044717	0.458450
H	-0.145314	0.136631	-2.332195
H	1.806062	1.579063	-1.591474
H	1.486191	2.473366	1.317366
H	3.928753	2.119775	1.149068
H	3.596223	3.694940	0.432357
H	-3.925088	3.822395	2.012754
H	-2.646522	4.302226	0.870812
H	-2.259603	3.244015	2.267561
H	-5.971742	-0.124578	-1.417446
H	-4.558555	-0.219182	-2.505365
H	-5.116668	1.374854	-1.880685
C	0.217535	-1.291073	0.776259
C	1.317975	-1.139000	1.543979
C	2.639045	-0.939485	0.979796
C	2.852589	-0.976513	-0.367938
C	1.739521	-1.110772	-1.290779
C	0.316037	-1.171430	-0.723741
C	-1.067704	-1.575200	1.429815
C	-2.065248	-2.316203	0.932961
C	-3.346229	-2.581932	1.672256
O	-4.482136	-2.367669	0.868804
O	3.704892	-0.684574	1.758311
C	3.548564	-0.537122	3.160434
O	4.081470	-0.628186	-0.855207
C	4.764776	-1.623117	-1.619302
O	1.898482	-1.090558	-2.506568
H	-4.410328	-1.498233	0.440890
H	1.217051	-1.223185	2.620044
H	-0.158152	-2.037964	-1.201403
H	-1.174002	-1.173806	2.438565
H	-2.006338	-2.750903	-0.062514
H	-3.377190	-1.975839	2.591870
H	-3.384568	-3.635362	1.973245
H	4.535269	-0.265083	3.533598
H	3.233941	-1.474695	3.630599
H	2.837387	0.259814	3.402647

H	5.715996	-1.176818	-1.910229
H	4.186661	-1.888111	-2.507289
H	4.947900	-2.507922	-0.999667

C6–C2′ dimer

C	0.146966	-0.677923	1.490069
C	0.567987	-1.379989	0.222127
C	2.046405	-1.343268	-0.059508
C	2.893274	-0.549650	0.637136
C	2.385094	0.321511	1.713825
C	1.004221	0.106018	2.163554
C	-1.244327	-0.889841	1.922532
C	-2.055849	0.072107	2.371424
C	-3.499219	-0.154814	2.725831
O	-4.361986	0.622123	1.915966
O	2.321842	-2.120622	-1.116548
C	3.648992	-2.333486	-1.595728
O	4.187306	-0.340021	0.225699
C	5.206070	-0.694457	1.161345
O	3.100847	1.183483	2.217703
H	-4.415291	0.209422	1.040764
H	0.280458	-2.437339	0.290011
H	0.714720	0.612618	3.078853
H	-1.642203	-1.895575	1.788919
H	-1.703749	1.102355	2.422906
H	-3.737919	-1.224326	2.652489
H	-3.684983	0.160685	3.757968
H	4.070167	-1.417016	-2.010242
H	4.294351	-2.711551	-0.798848
H	3.543819	-3.089371	-2.373749
H	5.165714	-1.771536	1.364331
H	6.154498	-0.446503	0.683898
H	5.094981	-0.128500	2.088251
C	-0.141942	0.647757	-1.205978
C	-1.249752	1.415341	-1.227008
C	-2.574853	0.853058	-1.042293
C	-2.778412	-0.493867	-0.931686
C	-1.664848	-1.419032	-0.942502
C	-0.246530	-0.845643	-1.022295
C	1.203372	1.205902	-1.389608
C	1.643489	2.338324	-0.833943
C	3.068651	2.817673	-0.921162
O	3.944224	1.908951	-1.543994
O	-3.661186	1.636157	-0.976259

C	-3.520076	3.010915	-0.640743
O	-4.017601	-0.949215	-0.578210
C	-4.697637	-1.740122	-1.550978
O	-1.815325	-2.632557	-0.826493
H	4.154062	1.204547	-0.910407
H	-1.155055	2.476212	-1.427310
H	0.212781	-1.325448	-1.896316
H	1.912056	0.604677	-1.955814
H	0.980512	2.934462	-0.207059
H	3.107191	3.748679	-1.500019
H	3.406311	3.057713	0.097450
H	-3.107541	3.585002	-1.476768
H	-4.528732	3.360185	-0.425544
H	-2.899952	3.132214	0.252035
H	-5.664128	-1.992146	-1.113588
H	-4.849670	-1.162466	-2.469699
H	-4.133819	-2.652469	-1.761908

C2–C2′ dimer

C	-0.526822	2.135547	-0.370287
C	0.206952	0.863366	-0.696948
C	-0.607783	-0.160159	-1.442491
C	-1.960847	-0.100289	-1.524737
C	-2.668881	1.055362	-0.958985
C	-1.864182	2.192366	-0.490467
C	0.218419	3.314766	0.082040
C	1.550965	3.439434	0.152148
C	2.239695	4.689649	0.615284
O	3.204386	5.142293	-0.320366
O	0.201317	-1.102752	-1.936666
C	-0.252629	-2.429183	-2.220235
O	-2.687926	-1.024162	-2.218081
C	-3.534005	-1.859136	-1.428869
O	-3.900008	1.101402	-0.903884
H	2.757789	5.344798	-1.147996
H	1.062765	1.116557	-1.331576
H	-2.424113	3.088965	-0.239355
H	-0.401864	4.163215	0.372136
H	2.212377	2.624459	-0.137541
H	1.502929	5.473088	0.843085
H	2.802640	4.489024	1.532327
H	-0.775171	-2.840430	-1.352232
H	-0.902642	-2.443708	-3.094612
H	0.657633	-3.001738	-2.394806

H	-4.225104	-1.267057	-0.825921
H	-4.093412	-2.475457	-2.133036
H	-2.929706	-2.506134	-0.778513
C	-0.184609	-0.646736	1.355653
C	0.825571	0.190107	0.607967
C	2.102148	-0.528738	0.254241
C	2.256056	-1.867256	0.383603
C	1.152393	-2.682280	0.928660
C	-0.002727	-1.970045	1.496305
C	-1.356513	0.049747	1.908763
C	-2.589317	-0.467280	1.947920
C	-3.801007	0.243304	2.481852
O	-4.912069	0.127417	1.621640
O	2.985141	0.357553	-0.242153
C	4.348880	0.022728	-0.498873
O	3.350288	-2.516502	-0.109785
C	4.124899	-3.237094	0.844810
O	1.203048	-3.908527	0.931695
H	-4.712719	0.570636	0.782212
H	1.118131	1.019373	1.263590
H	-0.701946	-2.583371	2.056239
H	-1.197675	1.070329	2.255378
H	-2.777157	-1.455906	1.530478
H	-3.557338	1.295560	2.694039
H	-4.107454	-0.217910	3.427411
H	4.430533	-0.703707	-1.306448
H	4.819032	0.966202	-0.776678
H	4.822072	-0.373659	0.403800
H	4.512683	-2.553866	1.612414
H	3.536090	-4.030223	1.310526
H	4.958281	-3.671888	0.291757

C2–O5′ dimer

C	-0.051594	2.258480	-0.041411
C	1.226388	2.825772	-0.103031
C	2.346310	2.067664	0.240003
C	2.195455	0.720566	0.609653
C	0.917581	0.157282	0.654561
C	-0.198786	0.928334	0.345069
C	-1.234459	3.061942	-0.406931
C	-2.470409	2.863892	0.062362
C	-3.682416	3.625502	-0.383077
O	-4.682683	2.753918	-0.892315
O	3.619429	2.527035	0.233805

C	3.845103	3.864027	-0.164208
O	3.309340	-0.033442	0.847653
C	3.587551	-0.290820	2.222832
O	0.838825	-1.155392	1.034875
H	-4.305073	2.246851	-1.618598
H	1.330769	3.861826	-0.403596
H	-1.180655	0.477070	0.363702
H	-1.063995	3.871781	-1.117122
H	-2.656948	2.084438	0.800247
H	-4.153135	4.134483	0.463308
H	-3.404722	4.389316	-1.123122
H	4.921365	4.017311	-0.091683
H	3.523429	4.031853	-1.199025
H	3.332017	4.570279	0.499747
H	2.748791	-0.814733	2.691640
H	4.475796	-0.923525	2.239355
H	3.790141	0.648668	2.748058
C	0.156300	-1.877365	-1.192109
C	-0.014617	-2.042510	0.300965
C	-1.441334	-1.972931	0.807536
C	-2.503745	-1.787412	-0.010744
C	-2.291611	-1.581163	-1.461623
C	-0.912447	-1.652485	-1.973615
C	1.507540	-1.939046	-1.751623
C	2.603333	-2.349211	-1.101600
C	3.983350	-2.333838	-1.688696
O	4.909148	-1.715637	-0.818056
O	-1.451528	-2.167750	2.132349
C	-2.526262	-1.690398	2.938715
O	-3.781185	-1.887793	0.453590
C	-4.594345	-0.720343	0.330950
O	-3.230681	-1.374550	-2.222195
H	4.476642	-0.950841	-0.408289
H	0.357996	-3.033636	0.587948
H	-0.810906	-1.528280	-3.047798
H	1.597115	-1.607754	-2.785484
H	2.548349	-2.665477	-0.062358
H	4.342826	-3.358303	-1.839633
H	3.966777	-1.840347	-2.671933
H	-2.745610	-0.645063	2.696962
H	-3.423301	-2.294953	2.806975
H	-2.161534	-1.758609	3.963510
H	-5.528734	-0.949544	0.844068
H	-4.114380	0.137865	0.818048
H	-4.789650	-0.487412	-0.716443
